# Enhanced cardiac perception predicts impaired performance in the Iowa Gambling Task in patients with panic disorder

**DOI:** 10.1002/brb3.206

**Published:** 2014-01-22

**Authors:** Julian Wölk, Stefan Sütterlin, Stefan Koch, Claus Vögele, Stefan M Schulz

**Affiliations:** 1 Department of Psychology I, University of WürzburgWürzburg, Germany; 2 Schön Klinik Roseneck, Hospital of Behavioral MedicinePrien am Chiemsee, Germany; 3 Lillehammer University College, Department of PsychologyLillehammer, Norway; 4 Research Group on Health Psychology, University of LeuvenLeuven, Belgium; 5 Paracelsus Medical University SalzburgAustria; 6 Research Group Self-Regulation and Health, Research Unit INSIDE, University of LuxembourgLuxembourg, Luxembourg; 7 Comprehensive Heart Failure Center, University of WürzburgWürzburg, Germany

**Keywords:** Cardiac perception, decision making, interoception, Iowa Gambling Task, panic disorder, somatic marker hypothesis

## Abstract

**Objective:**

Somatic marker theory predicts that somatic cues serve intuitive decision making; however, cardiovascular symptoms are threat cues for patients with panic disorder (PD). Therefore, enhanced cardiac perception may aid intuitive decision making only in healthy individuals, but impair intuitive decision making in PD patients.

**Methods:**

PD patients and age-and sex-matched volunteers without a psychiatric diagnosis (*n* = 17, respectively) completed the Iowa Gambling Task (IGT) as a measure of intuitive decision making. Interindividual differences in cardiac perception were assessed with a common mental-tracking task.

**Results:**

In line with our hypothesis, we found a pattern of opposing associations (Fisher's *Z* = 1.78, *P* = 0.04) of high cardiac perception with improved IGT-performance in matched control-participants (*r* = 0.36, *n* = 14) but impaired IGT-performance in PD patients (*r* = −0.38, *n* = 13).

**Conclusion:**

Interoceptive skills, typically assumed to aid intuitive decision making, can have the opposite effect in PD patients who experience interoceptive cues as threatening, and tend to avoid them. This may explain why PD patients frequently have problems with decision making in everyday life. Screening of cardiac perception may help identifying patients who benefit from specifically tailored interventions.

## Introduction

The effects of physical sensations on overt behavior have been subject to extensive research, often based on the somatic marker hypothesis (SMH, Damasio et al. 1991; Bechara et al. 1994; Damasio 1995). The SMH suggests, for example, that somatic cues guide decision making in complex situations, which are characterized by little explicit information to base a decision on, and/or time pressure. More specifically, the SMH posits, that responses in such situations are associated with specific, learned somatic states (e.g., heart rate, skin conductance, muscle tone), which were previously evoked by similar decisions. These “emotional marker” signals are represented in the anterior insular cortex and embedded in decision-making processes via ventromedial prefrontal pathways (Damasio 1995).

An established paradigm for the assessment of intuitive decision-making patterns under time pressure and with incomplete information is the Iowa Gambling Task (IGT, Bechara et al. 1994). Implicit learning skills have been reported to be positively associated with IGT performance (Bechara et al. 1997). The main body of literature has considered skin conductance response as a proxy for visceral somatic markers (Dunn et al. 2006), although the SMH suggests that cardiac cues may play a similar role. In line with this assumption, interindividual differences in trait cardiac perception accuracy have been found to affect emotional bias on speeded reactions in healthy volunteers (Sütterlin et al. 2013). Moreover, at least one study has demonstrated that healthy participants with particularly high cardiac perception outperform those with lower accuracy in the IGT (Werner et al. 2009).

The perception and cognitive evaluation of physical symptoms is considered to play a crucial role in the development and maintenance of panic disorder (PD). The psychophysiological model of PD (Ehlers and Margraf 1989) describes a vicious circle of perception of physical cues and their catastrophizing evaluation, which increases the probability of panic attacks. While healthy individuals attribute the experience of physical changes (e.g., beating heart, shortness of breath, etc.) to a variety of internal or external stressors, patients with PD habitually associate such sensations with imminent threat (Clark et al. 1988; Hofmann et al. 2008).

Increased sensitivity to physical cues (Barsky 1992; Ehlers and Breuer 1996; Eley et al. 2004; Hoehn-Saric et al. 2004) and their catastrophizing appraisal are typical features of PD and are often principal targets for PD treatment (Hofmann et al. 2008). A large body of research further supports the role of biased perception and interpretation of physical symptoms in the development (Bouton et al. 2001) and maintenance of PD (Ehlers 1993; Richards et al. 2003). There is evidence for increased perception of physical symptoms in PD patients (Domschke et al. 2010). Physical symptom perception is often part of PD patients' reported symptomatology (Zoellner and Craske 1999) with cardiac symptoms such as heart rate playing a prominent role (Hartl 1995).

In addition to increased symptom perception and its biased attribution to impending threat, there is also evidence for intolerance of uncertainty in patients with PD (Carleton et al. 2012; Mahoney and McEvoy 2012), reduced risk-taking behavior (Giorgetta et al. 2012), increased latency in speeded decision making (Kaplan et al. 2006) and heightened sensitivity to errors (Ludewig et al. 2003). Yet, whether increased perception of physical cues would impact upon intuitive decision making in PD patients remains unclear. Given PD patients' habitual catastrophizing interpretation of physical cues, it could be argued that increased interoceptive awareness is detrimental for intuitive decision making in patients with PD.

The aim of this study was to examine the effects of increased perception and processing of somatic markers on decision-making processes in PD patients. In line with previous studies (Domschke et al. 2010; Grosche et al. 2011), we expected to find enhanced cardiac perception in PD patients as compared to matched controls without psychiatric diagnosis. In control participants we further hypothesized cardioceptive perception accuracy to be positively associated with performance in intuitive decision making. In PD patients, we expected to find the opposite pattern of results, that is high cardioceptive accuracy to impair intuitive decision making due to PD patients' catastrophic interpretation of such cues.

## Methods

### Sample

The patient sample consisted of 17 inpatients (eight female patients; *M* = 41.59 years, SD = 13.30), admitted to a psychosomatic hospital (Schön Klinik Roseneck, Hospital of Behavioral Medicine, Prien, Germany). All patients had a principal diagnosis of PD as assessed by trained clinical psychologists. Diagnoses were based on DSM-IV-TR criteria (American Psychiatric Association 2000), and verified by J. W., based on the PD-related parts of the SKID-I (Wittchen et al. 1997). The control group comprised of 17 volunteers (eight female volunteers; *M* = 36.53 years; SD = 12.10) without mental disorders, matched for gender and age. As indicated by self-disclosure, none of the control participants had a psychiatric diagnosis or any cardiac and/or neurological disorder and, therefore, no related medication. Moreover, none of the participants had taken benzodiazepines within 2 weeks prior to the experimental assessment. Patients with an additional diagnosis of somatoform disorder were excluded, due to the suggested role of abnormal perception of physical symptoms in this diagnostic category. Nevertheless, those meeting criteria for secondary anxiety disorders (social phobia *n* = 1, 11.76%), generalized anxiety disorder (*n* = 1, 5.88%), or major depression (*n* = 16, 94.10%) were included, thus representing a typical sample of PD patients in clinical practice (Kaufmann and Charney 2000; Brown et al. 2001).

### Materials

#### Equipment

ECG was recorded with the NeXus-10® system (Mind Media BV/Roermond-Herten, Netherlands) using Einthoven lead I configuration with Ambu® Blue Sensor VL (Ambu GmbH/Bad Nauheim/Germany) electrodes. Data were sampled at 512 Hz. A freeware IGT application was run on a personal computer (ASUS®, Taipeh, Taiwan) with Windows XP operating system and presented on a 15-inch LCD-monitor at 1024 × 768 pixel screen resolution with ˜40 cm head-to-screen distance.

#### Iowa Gambling Task

The IGT consists of four decks of cards (A, B, C, D). Drawing cards from deck A or B results in large gains but high losses, leading to an overall loss. In comparison, drawing cards from decks C and D results in small gains but similarly small losses and an overall net profit (see Table 1 for details). Participants are instructed to draw 100 cards from these decks, with the aim to maximize their profit. Typically, control participants begin by selecting cards more or less randomly, followed by a period of implicit learning with a preference for the net gain option and finally explicit knowledge resulting in a clear preference for decks C and D.

**Table 1:** Characteristics of the Iowa Gambling Task.

	Deck A	Deck B	Deck C	Deck D
Gain per draw	100	100	50	50
Batch of possible losses per draw	{0,0,0,0,0,150,200,250,300,350}	{0,0,0,0,0,0,0,0,0,1250}	{0,0,0,0,0,0,0,0,0,55,55,55,55,55}	{0,0,0,0,0,0,0,0,0,250}
Range of net loss versus gain per draw	−250 to +100	−1150 to +100	−5 to +50	−200 to +50
Probability of positive net gain per draw	0.5	0.9	0.5	0.9

Each time a card is drawn from one of the decks, the associated gain is won but counterbalanced by a potential loss that is selected at random from the respective batch of losses. The net amount per draw results from subtracting the loss from the gain. Furthermore, achieving a positive net gain only occurs at a certain probability.

#### Mental-tracking task

Following Herbert et al. (2012), the participants were instructed to quietly count the heartbeats, which they experienced in the time interval between a start and a stop signal without any supplementary aids such as taking their pulse or estimating the expected number of heartbeats based on the (estimated) elapsed time. This task was performed for four time intervals with 20, 25, 35, and 45-sec duration and a 60-sec rest time between the time intervals. During this procedure, participants were asked to close their eyes, to sit relaxed, and to breathe consistently. Start and stop of each interval was indicated verbally by the experimenter. Particularly in small samples, randomization often does not produce comparable distributions of conditions across groups. Hence, the order of time intervals was not randomized, to increase procedural comparability between the two groups. Importantly, the individual participants were not aware of the fixed order. Instructions were given in written form to standardize the instruction (Ehlers et al. 1995). We added an initial warm-up trial to allow sufficient time for the transition from the instructional phase to the different mode of processing during interoception. The warm-up trial was not included in the analysis, as we aimed for an optimal compromise between keeping the scores comparable with previous reports as much as possible and the added benefit of reducing task-irrelevant training effects in a situation unfamiliar for most participants (Sütterlin et al. 2013).^1^

#### Self-report data

The German version of the State–Trait Anxiety Inventory (Laux et al. 1981) was used to assess habitual trait and state anxiety on two scales comprising 20 items each. Items are rated from 0 (not at all) to 3 (very much so), resulting in a scale range of 0–60 for each measure. Higher scores indicate higher anxiety. Cronbach's *α* is about 0.90 for both scales (Laux et al. 1981). The current sample achieved a Cronbach's *α* of 0.92 for the STAI-State and 0.96 for the STAI-Trait.

The German 21-item version of the Beck Depression Inventory (BDI) was used to assess dysphoric mood and depression. Items are rated on a four-point scale from 0 to 3, resulting in an overall score ranging from 0 to 63. Higher scores represent more severe symptoms of depression. Internal consistency is good with Cronbach's *α* = 0.89 (Hautzinger et al. 2006).

Trait anxiety sensitivity was assessed with the German version of the Anxiety Sensitivity Index (ASI, Peterson and Plehn 1999). Notably, high scores on the ASI have been shown to predict both the frequency of panic attacks in PD patients and increased cardioceptive accuracy (Domschke et al. 2010). Cronbach's *α* of the ASI has been reported to exceed at least 0.75 (Peterson and Plehn 1999); the current sample achieved an internal consistency of 0.95.

Positive and negative affect were assessed with the German version of the Positive and Negative Affect Schedule (PANAS, Krohne et al. 1996), assessing positive and negative affect on separate scales comprising 10 items each. Items are rated from 1 = “little” to 5 = “most” and each scale score ranges from zero to 50. Higher scores represent higher positive/negative affect. Cronbach's *α* is 0.85 for both scales (Krohne et al. 1996). The current sample achieved a Cronbach's *α* of 0.79 for the PA subscale and 0.88 for the NA subscale.

In addition, the PD group completed the Panik und Agoraphobie Skala (PAS, Bandelow 1997), which measures severity of PD on four scales comprising two items (panic attacks, agoraphobic avoidance, constraints in daily life and worry on healthiness), one scale with three items (anticipatory anxiety), plus one additional item to assess whether panic attacks are mostly unexpected or related to feared situations. The PAS overall score ranges from 0 to 52 with a cutoff of 9 indicating slight PD. Psychometric properties are overall sound with a test–retest reliability of 0.73, Cronbach's *α* of 0.86 and high convergent validity ranging from 0.58 to 0.76 (Bandelow 1997). Cronbach's *α* for the individual subscales ranged between 0.70 and 0.94 in the current sample.

There are reports on associations between performance in the mental heartbeat-tracking task and participant's gender (Ludwick-Rosenthal and Neufeld 1985) and body mass index (BMI, Montgomery et al. 1984; Jones et al. 1987), as well as between educational level and IGT performance (Davis et al. 2008). We assessed gender and educational level as control variables via self-report questionnaires. BMI was assessed at a medical examination during admission.

### Procedure

The study was carried out in compliance with the Code of Ethics of the World Medical Association (Declaration of Helsinki) and was approved by the ethics committee of the Schön Klinik Roseneck. Participants participated voluntarily and received no compensation for taking part in the study. Signed informed consent was obtained for subjects after the nature of the procedures was explained. Next, participants completed the questionnaires and subsequently performed the mental-tracking task. After assessment of cardiocepetive accuracy, the participants performed the IGT.

### Data reduction

**Figure 1:**
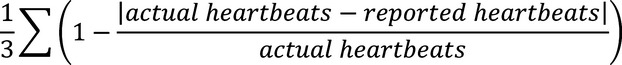
Formula for computing the cardioceptive accuracy index across three time intervals (Werner et al. 2009).

First, interbeat intervals were extracted from the raw ECG using ARTiiFACT (Kaufmann et al. 2011). Next, for each time interval of 25-, 35-, and 45-sec cardioceptive accuracy was calculated with the formula presented in Figure 1. The result is an index that ranges from 0 to 1, with 1 indicating perfect accuracy of heartbeat detection.

### Statistical analysis

All data were checked for normal distribution with Kolmogorov–Smirnov tests and Lilliefors Significance Correction. The various group characteristics were compared using independent sample *t*-tests. To assess associations of cardioception with IGT parameters and other variables, Pearson's correlations were computed. Group differences in cardioceptive accuracy and IGT were compared with independent samples *t*-test. Fisher's *Z* test for independent samples was used to compare correlations between cardioceptive accuracy and IGT performance in both groups.

A priori sensitivity analysis (G*Power 3.1) resulted in a critical *r* = 0.48 to achieve test power of 0.80 (*α* = 0.05) in two-tailed bivariate testing given the available sample size. Varying group sizes in single tests may occur due to missing items in self-report data.

## Results

### Sample characteristics

Patients with PD did not differ from matched controls in terms of age, BMI, educational level, and state and trait anxiety. Significant differences between groups, however, occurred for positive and negative affect, depression, and anxiety sensitivity (see Table 2 for details). Depression and anxiety sensitivity did not correlate with other variables in both groups. Both groups consisted of nine male and eight female participants, their educational level was high. Of these, 58.8% of PD patients and 58.9% of control participants reached a higher education entrance qualification. Both groups were comparable with regard to physical activity.

**Table 2:** Means (*M*), standard deviations (SD), or frequencies (*n*) and percent (%) significance level (*P*) and effect size (Cohen's *d,* calculated on basis of control group's standard deviation) of group characteristics for patients with panic disorder (PD) versus matched controls.

	PD patients		Matched controls				
	*M* or *n*	(SD or %)	*M* or *n*	(SD or %)	Test statistic	*P*	Effect size
Age	41.59	(13.26)	36.53	(12.61)	*t*(32) = −1.14	0.263	*d* = 0.40
BMI (kg/m^2^)	24.58	(2.69)	23.70	(3.06)	*t*(31) = −0.87	0.383	*d* = 0.29
Educational level^1^							
Hauptschule (2)	2	11.76	2	11.76	*V*(*df*) = 0.09	0.966	*Ф* = 0.09
Realschule (2)	5	29.41	5	29.41			
(Fach-)Abitur (3)	3	17.65	2	11.76			
Hochschulabschluss (5)	7	41.18	8	47.06			
Participants practicing any kind of sports (in all cases this included endurance training)	12	70.59	14	82.35	Fisher's exact test	*P* > 0.069	*–*
Frequency of training per week	2.67	.88	2.71	1.33	*t*(24) *=* 0.11	0.92	*d =* 0.04
Duration of training per week	1 h 32 min	2 h 21 min	4 h 24 min	7 h 9 min	*t*(24) *=* 0.11	0.92	*d =* 0.04
STAI-state	47.19	(3.71)	49.88	(3.26)	*t*(31) = 2.22	0.034*	*d* = 0.83
STAI-trait	47.41	(2.92)	49.59	(2.94)	*t*(32) = 2.17	0.038*	*d* = 0.74
Positive affect	22.47	(5.03)	30.59	(2.91)	*t*(32) = 5.76	<0.001***	*d* = 1.98
Negative affect	19.06	(6.92)	10.88	(0.93)	*t*(32) = −0.83	<0.001***	*d* = 1.66
BDI	34.43	(7.92)	3.40	(3.60)	*t*(27) = −13.74	<0.001***	*d* = 8.62
ASI	35.47	(12.90)	9.94	(6.40)	*t*(32) = −7.31	<0.001***	*d* = 3.99

BMI, body mass index (kg/m^2^); STAI, state trait anxiety inventory; ASI, anxiety sensitivity index; Hauptschule, Realschule, and (Fach-)Abitur can be considered different levels of high school degrees (in ascending order).

1 In parentheses: international standard classification of education (ISCE) according to the UNESCO guidelines from 2011.

* *P* < 0.05

*** *P* < 0.001.

### Cardioceptive perception

In contrast to our expectations there were no significant group differences in scores of cardiac perception (*t*(28) = 0.85, *P* = 0.20, *d* = 0.31). PD patients showed a tendency for lower accuracy (*M* = 0.61, SD = 0.20) as compared to matched controls (*M* = 0.68, SD = 0.22).

### IGT performance

On average, PD patients chose a successful card in 46.79 trials out of 100 trials (SD = 8.61, min = 29, max = 64), as compared to the matched controls, selecting winning cards in 48.31 trials out of 100 trials (SD = 11.88, min = 24, max = 65). More precisely, the PD group chose deck B most of the time followed by decks D, A, and C. The control group chose deck B most often, followed by decks D, C, and A. For details see Table 3. Nevertheless, in contrast to previous studies, participants did not switch their strategy after an initial period from exploration of all decks to selective preference of decks with a positive yield (C and D), but continued switching decks unpredictably throughout the experiment.

**Table 3:** Means (*M*), standard deviations (SD), minimum (Min), and maximum (Max) of cards drawn from the four decks (A, B, C, D) for the panic disorder (PD) patient group as compared to matched controls.

	PD patients				Matched controls			
Deck	*M*	SD	Min	Max	*M*	SD	Min	Max
A	23.71	6.30	12	33	16.44	10.10	3	41
B	29.50	10.80	11	53	35.25	11.70	16	53
C	20.57	5.80	9	29	20.31	11.60	6	37
D	26.21	7.60	15	40	28.00	14.50	5	56

### Associations between cardioceptive skill and IGT performance

In line with our hypothesis, the correlations between cardioceptive accuracy and IGT performance differed significantly between groups (Fisher's *Z* = 1.78, *P* = 0.04).

**Figure 2:**
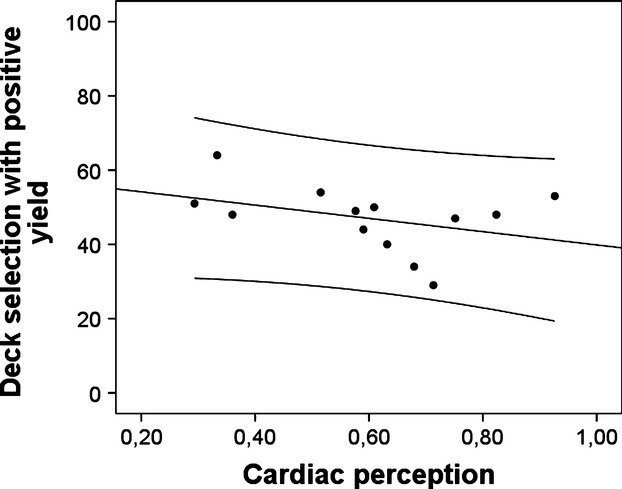
Scatter plot for correlations of cardioceptive skill with IGT performance in the group of PD patients, including linear regression line plus lines for margins of one standard deviation.

**Figure 3:**
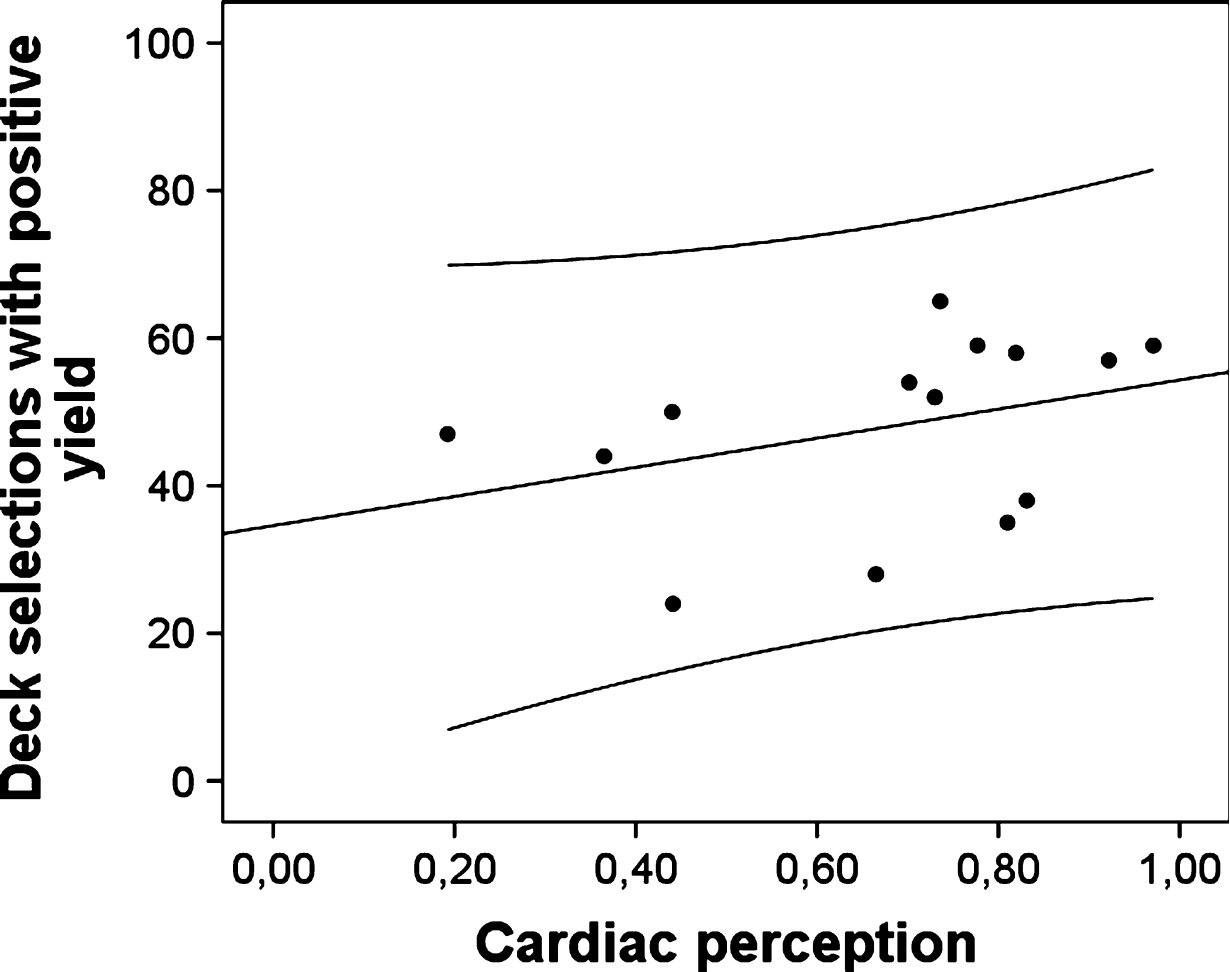
Scatter plot for correlations of cardioceptive skill with IGT performance in the control group, including linear regression line plus lines for margins of one standard deviation.

When tested against zero, there was a positive but not significant correlation between cardioceptive accuracy score and positive deck selection in the control group (*r* = 0.36, *n* = 14, *P* = 0.10; see Figure 2), and an almost equally strong negative correlation in the PD group (*r* = −0.38, *n* = 13, *P* = 0.10; see Figure 3), suggesting that PD patients with high-cardioceptive accuracy selected advantageous decks less often.

Cardioceptive accuracy did not correlate significantly with depression (*r* = −0.45, *n* = 12, *P* = 0.14), state anxiety (*r* = −0.27, *n* = 14, *P* = 0.36) or trait anxiety in the PD group (*r* = −0.01, *n* = 15, *P* = 0.97) or the control group (depression: *r* = 0.37, *n* = 13, *P* = 0.21; state anxiety: *r* = 0.37, *n* = 15, *P* = 0.18; trait anxiety: *r* = 0.01, *n* = 15, *P* = 0.97).

## Discussion

In this study, we investigated whether cardioceptive accuracy in patients with PD predicts performance in a complex decision task, requiring implicit and explicit learning (i.e., the IGT). In line with predictions derived from somatic marker theory (Damasio et al. 1991), we expected that utilization of interoceptive cues aids intuitive decision-making in age and sex-matched control participants without psychiatric diagnosis. However, in PD patients we expected the opposite, because interoceptive information, in particular when related to cardiac symptoms, comprises a major source of threat to them, sometimes triggering panic attacks (Ehlers and Margraf 1989; Hofmann et al. 2008). Therefore, rather than utilizing interoceptive – in particular cardioceptive information, we assumed that PD patients may rather try to avoid it, hence being distracted rather than guided by somatic cues. Therefore, we expected that high-cardiac perception, would rather impair decision making, and hence, IGT performance in PD patients. In line with our hypothesis, we found significantly different and opposing patterns of association between cardioceptive accuracy^2^ and IGT performance in patients with PD and matched controls.

Control participants tended to benefit from increased cardioceptive accuracy in terms of better IGT performance. First, this replicates evidence for an association between enhanced cardiac perception and intuitive decision making (Werner et al. 2009). Second, this is in line with a study indicating that enhanced cardiac perception is associated with avoidance of seemingly risky choices in a framing task, where trials with objectively equal options were framed emotionally by suggesting that one would either have the chance to *win* or face the risk to *lose* an equal amount of money in a given trial (Sütterlin et al. 2013).

This is the first study to show that cardiac perception can also have the opposite effect: In PD patients, the association of high-cardiac perception with IGT-task performance was significantly different. High cardiac perception rather impaired than supported intuitive decisions in the IGT. This result suggests qualitative differences between control participants and PD patients in the processing of interoceptive information.

It could be argued that enhanced cardiac perception may feed into dysfunctional cognitive appraisal. This can be well integrated into classical vicious-circle models of PD (Ehlers and Margraf 1989). According to these models, perception of symptoms leads to catastrophic interpretations, thus increasing autonomic arousal and physical symptoms that can be perceived as threatening. Avoidance of associated eliciting cues then leads to generalization and maintenance of PD. In complex decision-making tasks this may have detrimental effects, when attention to associated information is withdrawn due to generalized avoidance of somatic cues as described in the SMH.

Future studies should examine real-life decision making in PD patients based on such models. This could well lead to better explanations why PD patients' history is often characterized by decision difficulties (Ludewig et al. 2003; Lorian and Mahoney 2012).

Although the group difference was not significant, controls exercised about twice as long per week as panic patients. Although cardiac perception was similar in both groups and the correlations with decision making were not stronger in the control group, this indicates that panic patients may be less familiar with experiencing cardiac symptoms in a safe context. In patients with high cardiac perception, this may further add to the presumed detrimental effect of experiencing cardiac somatic cues on decision making. From a clinical point of view, it may therefore be interesting to address such a potential association of (cardiac) somatic cues with panic-related (negative) associations. Symptom-focused exposure (e.g., elicited by physiological provocation tasks and discrimination learning) could help to weaken these associations. Once cardiac symptoms are not experienced as threatening anymore, this may also withdraw the basis for the side effects of (cardiac) somatic markers on decision making as delineated above. Screening for cardiac perception may help identify patients who may profit from such an approach.

### Limitations

First, it should be noted, that almost no patient reached the stage of explicitly understanding the effects of selecting a particular deck in the IGT. Therefore, our findings only apply to the stage where participants decide randomly or rely on a hunch. However, it is clear from somatic marker theory that this is the stage where cardioception would be considered to have the largest impact on behavior. Second, we did not find a main effect of group. This is at odds with previous studies suggesting that PD patients may generally have higher cardiac perception (for a review see, Domschke et al. 2010). However, not all previous studies have found this difference, and the small to medium effect size in our study could become significant in a larger sample. Comparing these studies, it is interesting that both participants recruited from inpatient versus outpatient settings occur. Maybe differences in symptom severity, comorbidity, treatment intensity, time since diagnosis, etc. contribute to a larger heterogeneity in our patient group, reducing effect size due to error variance. Future studies should therefore pay particular attention to such moderating factors. Third, it has been suggested that the mental-tracking task may reflect cognitive ability rather than interoceptive skill. Although the present task was designed to prevent the respective strategy of estimating the time passed during counting of heartbeats, we cannot completely rule out that cognitive ability is confounded with cardiac perception scores. Nevertheless, the reverse association of high-cardiac perception and decision-making performance found in panic patients would still be at odds with this alternative explanation. Moreover, it appears quite hard to find a plausible explanation why high-cognitive skill may predict impaired decision making. Hence, we consider our interpretation as the more parsimonious one. Fourth, the small sample size limits the generalizability of our results, therefore, warranting future replications in larger samples. An independent replication of these results with a larger sample size and consequently more heterogeneous sample (e.g., including typical comorbidities) could increase the generalizability of our conclusions. Nevertheless, several observations support the reliability of the current results. First, the correlations are similar to previously published results on interoceptive accuracy and anxiety (Pollatos et al. 2007; Domschke et al. 2010) or those achieved with a similar task (Sütterlin et al. 2013). Second, the sample was homogeneous and carefully selected to minimize effects of comorbidity and medication, and third the results do not depend on outliers, single participants or small groups of individuals (see Figs. 2, 3). Fifth, the test situation in the laboratory may have induced moderate stress in all participants. This may have induced particular attention to or salience of cardiac cues in some PD patients. On the other hand, a similarly stressful context is present in many real-life situations requiring decision making, hence, this could also be seen as a factor increasing the ecological validity of this study, improving generalizability of these findings to real-life situations.

## Conclusion

This is the first study to demonstrate a detrimental effect of enhanced cardiac perception on intuitive decision making in PD patients. These findings extend previous results indicating that PD patient experience heightened awareness and dysfunctional cognitive processing of somatosensory cues, particularly cardiac activity (Hofmann et al. 2008) and associated bias (Amrhein et al. 2005). Together with cognitive models comprising of a vicious circle involving vigilance-anxiety-avoidance (Clark et al. 1988), our findings provide new insight into the basis for detrimental decision making in PD patients.

## Notes

1 It may be noted that including these data in the analysis did not affect the results.

2 Please note the current discussion on related terminology and what exactly is assessed with the mental-tracking task (Ceunen et al. 2013).
